# The Art of Convalescence

**Published:** 1920-05-22

**Authors:** 


### THE PUBLIC WELL-BEING.

### THE ART OF CONVALESCENCE.

"To be sick," wrote Charles Lamb, from a convalescent couch, "is to enjoy monarchal prerogatives. Compare the silent tread, the quiet ministry, almost with the eye only, with which he is served—with the careless demeanour, the unceremonious goings in and out (slapping of doors, or leaving them open) of the very same attendants, when he is getting a little better—and you will confess that from the bed of sickness (throne rather let me call it) to the elbow chair of convalescence, is a fall from dignity, amounting to a deposition." Even the changed attitude of his medical attendant makes him, querulous and petulant. "Can this be he—this man of news—of chat—of anecdote—of everything but physic—can this be he, who so lately came between the patient and his cruel enemy, as on some solemn embassy from Nature, erecting herself into a high mediating party? Pshaw! 'tis some old woman." But I do not think Lamb's illness on this occasion (writes a correspondent) was very serious or excessively painful; and the milder the sickness the more peevish the convalescence. Hazlitt, the morose and irritable Hazlitt, revealed in his essay on "The Sick Chamber" a truer insight into the mental attitude of the patient who has "turned the corner" when he contrasted the fantastic misery of pain with the inexpressible relief of its sudden departure—the sense of calm, of rest, of vague pervasive happiness. "It is hardly to be wondered at," he says, "that we dread physical calamities so little beforehand; we think no more of them after they have happened. Out of sight out of mind. This will perhaps explain why all actual punishment has so little effect; it is contrary to the nature, alien to the will."

### Care-free Inanition.

There is sound sense in this point of view, and experience confirms it. I have undergone two dangerous illnesses, each of which contained a period of acute pain. In the first, when I was nearly carried off by peritonitis, my convalescence was slow and sure; in the second, an attack of appendicitis was followed by an unusually severe operation—in those days the removal of one's vermiform appendix was not so casual or so trivial an incident in the surgeon's working day as now it seems to be—and my recovery was fairly rapid. Both these rude interruptions of an otherwise healthy existence took place many years ago in early manhood, and the strange thing is that though my memory of the pain which I suffered is so remote and indistinct that I could not describe it if I wished to, any recollections of the two periods of convalescence are as fresh and agreeable as ever. In each the anguish of bodily suffering seemed suddenly to fall away, as if a heavy suffocating garment had been removed by magic, and left me in that state of care-free inanition which the Buddhists acclaim as near the highest heaven. Then I began to take a pensive if detached interest in the gentle attentions of the nurse. Weak as a babe but perfectly content, I allowed myself to be tenderly rolled on one side while the bed was made—an act of hygiene which roused the convalescent Lamb to staccato exasperation—and purred inwardly like a cat while my hands and face were caressed by a warm wet sponge. The stream of sunlight that flowed into the room and played pranks upon the counterpane—how new and wonderful it seemed! The twittering of the sparrows outside—how obvious and friendly their congratulations! The joy of the first cup of weak milk tea! The brief visits of members of the family, smiling gaily; the bunch of roses with their unspoken message from a girl friend in the white house down the road; that affectionate letter from a beloved sister at school in Scotland! The books, a score of them, lying on the little round table and awaiting my vivacious appetite—such a glorious adventure among my books I never had before or since. No, coming back to life from the torments of hell is not an unpleasant experience. It is like being born again, without pain, into a new and friendly world, with your senses in delicious languor but acutely receptive of impressions from which all that is distressful—and therefore likely to endanger your recovery—is decorously excluded by the skilful goddesses who watch over your delicately poised safety.

### A Hospital Drama.

It is perhaps a wise precaution, too. My second convalescence proceeded in the surgical ward of a famous London hospital, where an event occurred that "threw me back" a week—so the sister said—because it produced just that kind of impression which shocks the refugee from death out of his carefully encouraged egotism. The occupant of the bed next to mine was a jolly, middle-aged man who had been treated for a compound fracture of the left arm. He was able to dress and move about the ward, and was expecting to leave the institution after the last of a series of minor skin-grafting operations had been performed. One sunny afternoon, a few hours before the final operation was to take place, he came to my bedside in a state of nerveless terror, saying he had just fallen asleep and dreamed with frightful vividness of detail how he had struggled and died under the influence of an anæsthetic, and he was convinced that the dream foredoomed him in the coming operation. I tried to comfort him by pointing out that he had "gone under" five times already without any ill-effects, and that his fears were illogical and absurd. But he was right and I was wrong. That night, after the lights were lowered, the surgeon and the anæsthetist entered the ward and walked straight up to my neighbour's bed. For a few minutes all was silent. Then I heard from behind the red screen a gurgling gasp and a startled "What's

**THE PUBLIC WELL-BEING**—(*continued*).

that?" I knew instinctively and at once—the foolish dream was verified. For hours, it seemed to me, the surgeon and his partner worked like galley-slaves to bring him back to life; and nurses, obeying their curt requests, flitted like uneasy ghosts up and down the ward. But from the first I had no hope. He was as dead as he ever would be, and before the dawn they wheeled him silently away to the mortuary. That is why I was "thrown back" a week.

In all other respects it was an interesting and delightful convalescence, and the day the cab came to fetch me away to take my own post again unaided in the battle of life, I could have wept my regrets. It was like leaving a green oasis for the blazing desert. But always remember this: to enjoy your convalescence you must adopt the right attitude of mind towards the past, the present, and the future. Do not reflect and do not worry; just bask in the sensuous pleasures of inanition, or you will become restless and feverish and see a stern and anxious look in the gaze of your ministering angels. In order to draw a convincing conclusion from my own vicissitude in a bed of sickness it might be profitable to ascertain, from the point of view of, say, a dozen experienced nurses, the normal proportion of tractable to intractable convalescents. I believe the balance of evidence would be in my favour—and against Lamb.

